# Challenges in Job Seeking and the Integration of Ukrainian War Refugee Healthcare Workers Into the Polish Healthcare System: Facebook Content Analysis

**DOI:** 10.3389/ijph.2023.1606139

**Published:** 2023-09-05

**Authors:** Joanna Gotlib, Ilona Cieślak, Dominik Wawrzuta, Mariusz Jaworski, Dimitris Theofanidis, Grażyna Wójcik, Piotr Małkowski, Mariusz Panczyk

**Affiliations:** ^1^ Department of Education and Research in Health Sciences, Faculty of Health Sciences, Medical University of Warsaw, Warsaw, Poland; ^2^ Department of Nursing, International Hellenic University, Thermi, Greece; ^3^ Polish Nurses Association, Warsaw, Poland; ^4^ Department of Surgical, Transplant Nursing and Extracorporeal Treatment, Medical University of Warsaw, Warsaw, Poland

**Keywords:** barriers, Ukraine, refugee medical professionals, shortage, social media content

## Abstract

**Objectives:** The full-scale Russian invasion of Ukraine resulted in a refugee crisis. The terms of employment of Ukrainian Refugee background Medical Professionals (UKR-MPs) in the Polish healthcare system were liberalised. The aim of the study was to identify challenges in job seeking and the integration of Ukrainian war refugee healthcare workers into the Polish healthcare system.

**Methods:** A qualitative, descriptive study based on content thematic analysis of Facebook content. We analyzed 1,700 posts published on two public Facebook groups intended for UKR-MPs.

**Results:** The most common problems encountered by UKR-MPs were: 1) lack of easy-to-understand information about the list of documents necessary to apply for a work permit, 2) lack of feedback from those responsible for handling individual cases, and 3) long waiting time for the decision issued by the Ministry of Health.

**Conclusion:** Despite the promptly implemented solutions enabling access to the job market by UKR-MPs, the refugees have encountered considerable administrative difficulties, as well as those arising from insufficient knowledge of the regulations on working as medical professionals in Poland under the EU law.

## Introduction

The full-scale Russian invasion of Ukraine resulted in a refugee crisis, with the number of refugees exceeding 8 million people [1] and as reported by the UNHCR, the majority of them residing in Poland (1.5 million) [2]. Pursuant to the Temporary Protection Directive implemented by all EU member states [3], Poland passed the Act on Assistance to Ukrainian Citizens in Connection with the Armed Conflict on the Territory of that Country [4], regulating most issues related to the unprecedented number of refugees staying in Poland, including the terms of employment of Ukrainian Refugee background Medical Professionals (UKR-MPs).

Under the domestic and international law in force in Poland, a refugee needs to be granted refugee status under the 1951 Refugee Convention, and hold a refugee travel document [5]. In Poland, these regulations have now been relaxed to account for the possibility of receiving the UKR status by individuals who are war refugees from Ukraine, but are not considered refugees under the provisions of the Refugee Convention [4].

In the EU member states, the right to practice as a doctor or nurse is strictly regulated and has so far only been issued to individuals who 1) graduated from the respective studies or 2) had their diploma recognized in one of the EU countries.

Following Russia’s full-scale invasion of Ukraine in February 2022, the requirements for obtaining by UKR-MPs the right to practice medicine or nursing in Poland have been liberalized [6]. In Poland, the simplified procedure consists of two stages. Firstly, the applicant needs to obtain a licence to practice as a doctor/nurse in the territory of the Republic of Poland issued by the Minister of Health; secondly, the applicant needs to obtain a licence to practice as a doctor/nurse issued by the competent chamber of the professional self-government: medical or nursing. UKR-MPs with a medical or nursing diploma, a certificate of good health from an occupational medicine physician, a declaration of impeccable credentials and full legal capacity, and a document confirming citizenship, are granted permission by the Minister to practice as doctor or nurse in the territory of the Republic of Poland [4, 7]. To carry out the formalities, the applicant submits a set of documents, translated by a sworn translator into Polish, to the Ministry of Health. Having received approval from the Ministry of Health, the applicant needs to apply to a given District Chamber of Physicians or Nursing (depending on the planned place of employment) in order to have a specific work permit issued [8].

Despite the simplified work permit procedure; UKR-MPs may still face various challenges due to substantial differences in educational standards and their unawareness of varying standards of practice in Ukraine and in the EU member states. Insufficient knowledge of the Polish language may also constitute a major obstacle. To identify the challenges faced by UKR-MPs, arriving in Poland as a result of the refugee crisis and planning to work as doctors or nurses, we categorised the content created by Facebook users [9, 10].

The study was based on a well-documented theoretical framework. Over the past decade, an expanding literature has explored the ways in which refugees rely on mobile communication technologies and social media content to stay in touch with a wider community and to access relevant information and services in their new places [11]. There is emerging evidence that mobile phones and social media have become essential tools for accessing information and resources that can help refugees navigate their migration journeys and the complexities of life during resettlement. It is estimated for instance that 68% of refugees living in urban centers have access to an internet-enabled phone, with the vast majority prioritizing mobile ownership and connectivity as crucial for their successful adaptation [12-16].

The aim of the study was to identify challenges in job seeking and the integration of Ukrainian war refugee healthcare workers into the Polish healthcare system by categorising the content created by Facebook users.

## Methods

### Design

A qualitative, descriptive study based on Facebook content thematic analysis.

### Setting

The qualitative method used involves the process of perception, interpretation and conceptualisation of the meaning of qualitative data.

### Rigour

The criteria to determine rigour or trustworthiness in a qualitative study are credibility, dependability, conformability and transferability. Qualitative content analysis was performed according to Graneheim & Lundman [17] and instruction from Graneheim et al [18]. The method of category identification and annotation we used is based on the well-established methodology put forward by Broniatowski et al. [19].

### Study Context

The selection of thematic groups on Facebook was driven by their accessibility (public access) and the thematic focus of the group.

We focused on posts published on two, large public Facebook groups: “Doctors, Nurses from Ukraine - work” (since 11 March 2022; 1.8 thousand users) [8] and “Doctors and nurses from Ukraine in Poland” (since 9 July 2022; 777 users) [9], both launched and administered by Polish citizens.

These groups were selected for analysis as they are the only public groups available on Facebook aimed at particular users - doctors and nurses from Ukraine, arriving in Poland after 24 February 2022.

### Data Analysis

The Python programming language was used to access the Facebook Scraper library [20], through which all the posts were downloaded, along with the comments and number of reactions assigned to them, as well as the photos and videos published by the users. They were all machine translated into Polish using the DeepL translation tool [21].

The final dataset comprised a total of 3,038 user messages and the first stage of content analysis included categorizing data into: 1) Posts (835 items), 2) Comments on posts (1,142 items) and 3) Responses to comments on posts (1,061 items). Supplementary Material S1 presents the results of the search on Facebook.

**FIGURE 1:**
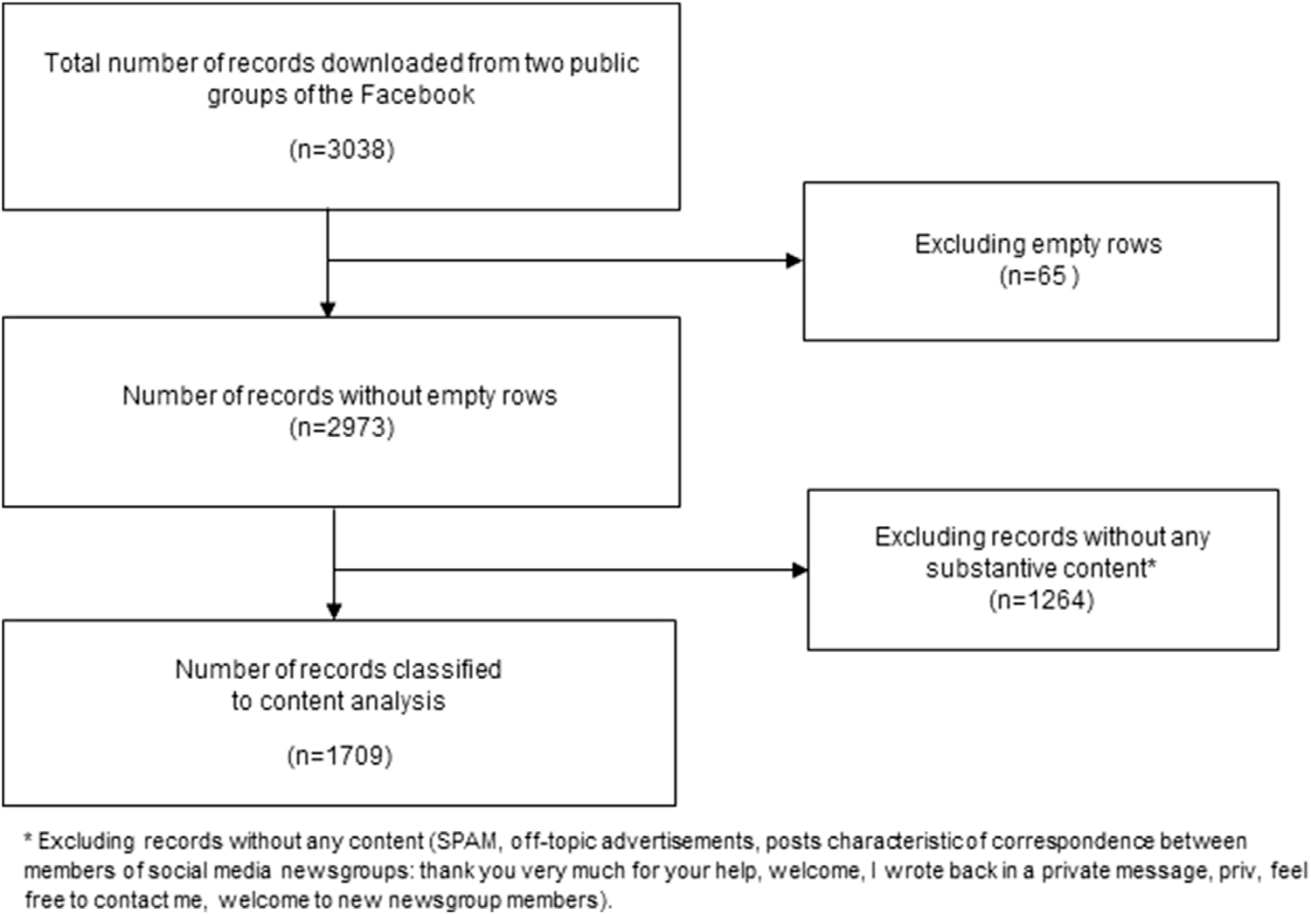
Flow diagram of Facebook posts selection (Poland, 2023).

The first stage of database optimization was to remove rows containing no content (empty rows, *n* = 65). Subsequently, data were deleted that did not constitute substantive content: SPAM, off-topic advertisements, posts characteristic of correspondence between members of social media newsgroups, e.g., thank you very much for your help, hello, I replied in a private message, priv, contact me, greetings to new members (*n* = 1,264). Thus, a total of 1,329 records were removed from the database. Later, the database of the remaining 1,709 machine translated records was proofread by a Ukrainian-Polish translator (Figure 1).

To ensure credibility, the authors spent an extended period of time on data collection and reviewed it repeatedly.

Content analysis involved six steps: getting acquainted with the data, initial coding, identifying themes, reviewing themes, formulating themes and assigning labels to themes. First, the posts were thoroughly read to gain familiarity with the data. Next, key contents were extracted and arranged into a preliminary code table. Similar codes were grouped together under the same codes. The codes were then categorized into potential subthemes and themes. The fourth step entailed reviewing and refining the themes to enhance their accuracy. In the fifth step, the themes were made more specific based on their nature and what they discuss. The last step involved labeling the themes based on their definition. By means of consensus, a codebook with six main categories and sixteen subcategories (Table 1) was established by two researchers (JG and IC).

**TABLE 1:** The codebook with six categories, 16 subcategories and example comments on challenges for Ukrainian Refugee background Medical Professionals entering the Polish healthcare system job market extracted from the Excel database (**Supplementary Material S1**) (Poland, 2023).

Categories (*n* = 1709)	Subcategories	Example comments
I. Course of the procedure for obtaining permission by UKR-MPs to work as doctors and nurses in healthcare entities in Poland (*n* = 661)	1. Lack of clear information regarding the list of documents necessary for the initiation and conduct of the procedure	- Where should I apply first and what my next steps should be? After graduating from university in Ukraine, I only have a diploma, what about an internship in Poland?
		- Is it necessary to translate the documents? And how should I send them?
		- What certificate of no criminal record did you submit? They phoned me and said that I should submit a certificate stating that I have no record of professional misconduct. And I only have a certificate from the Ministry of Internal Affairs of Ukraine that I have no criminal record (i.e., none) and am not a wanted person
	2. Lack of feedback on the course of the procedure from the MH	- Is there any way to find out the status of the work permit procedure being in progress?
		- I submitted the documents in person at the beginning of May, the confirmation of receipt has not come and the permit is not there either. Maybe someone has tried to speed it up through the employer, hospital?
	3. Long waiting time for the approval for work by the MH	- 6 months and 2 weeks
		- Yes they want to hire us but the employer has already found another person to take my place. Who will wait more than 3 months?
		- Thank you, I have been waiting for 4 months and I understand that I have to call, write. And I had naively hoped that everything was well set up and a decision would come quickly
		- Apparently the applications of those who applied in August have not even been processed yet (comment dated 14/12/2022)
II. Job offers for UKR-MPs (*n* = 443)	4. Job offers in healthcare entities in Poland for UKR-MPs	- Hello to the group. We are urgently looking for a doctor to work in a primary care outpatient clinic in a small town near Warsaw. We offer PLN 52 net per hour + accommodation free of charge
		- Hi, I work in a hospital. I am looking for doctors and nurses. The offer also covers board and lodging
		- Hi, we have a full-time opening, but only for a psychiatrist
		- Hi, I am looking for a dentist, orthodontist and dental assistant
	5. Advertisements of employment agencies for foreigners staying in Poland	- Urgently needed general practitioners, therapists, surgeons, anesthetists, pediatricians (ED physician, primary care physician, walk-in clinics physician, anesthetists) with decision-making capacity/ Physician’s licence for central district hospital, Maków Mazowiecki, Mazowieckie voivodeship. Accommodation provided for the first 3 months! If you have any questions, please contact us via private message [phone number] or e-mail [email address]
		- Are you looking for work? Are you a DOCTOR or NURSE maybe a DENTIST? Do you want to work in Poznań then you are welcome to join us:) We will help you with the paperwork to the MH and the District Chamber The salary and working conditions are discussed individually with More [phone number] [name of employment Agency] [email address]
	6. Job offers for UKR-MPs seeking work in other countries	- Urgent Employment for a Dentist to work in the UK. If interested please write YES or send me a message
		- Is there anyone here willing to travel as an elderly caregiver to Germany? Communicative German language required. Interested persons are welcome to contact me
		- Hello, I am looking for Ukrainian doctors who would like to work in France. I will get all the necessary documents
III. Searching for work by UKR-MPs in medical entities in Poland (*n* = 319)	7. Employer-employee relations	- I have obtained a residence card and a PWZ [right to practice my profession]. Under what conditions should I be employed: contract of employment or mandate agreement? My employer does not know, and I only have 7 days to inform the MH that I am employed, because they will cancel the work permit issued
		- Where can I find a link to some official source of information on employment conditions?
		- Do I need to have a certificate from the employer who will hire me?
	8. Knowledge of the Polish language	- Under the simplified employment procedure for Ukrainians, no proof of Polish language skills is required
		- I’m not working in the profession yet, they are saying everywhere that my language skills are insufficient
		- You need to know the language. I communicate with Poles, but at the moment it is not enough, unfortunately
	9. Looking for a job as a doctor	- Do you need an ultrasound diagnostics doctor?
		- I graduated from a medical university in Ukraine this year, I have a medical degree, I did not complete my internship. I have skills in acupuncture. I would like to work in Poland in the medical domain. My next steps - where to go to continue my studies or work, what documents do I need?
		- I am a dermatologist-therapist, cosmetologist from Ukraine. I have more than 10 years of work experience, all my diplomas and certificates are valid, I speak a little Polish at a conversational level. I am looking for work as a doctor or cosmetologist in Gdynia, Gdansk and the surrounding area
	10. Looking for a job as a nurse	- Is it possible to work as a nurse’s auxiliary until I receive a decree from the ministry?
		- I have two degrees: junior pharmacist and junior feldsher, I have worked as a nurse for 12 years, I have the first category, I am looking for a job
		- I have a diploma in nursing, but I have been working in the physiotherapy department as a therapeutic massage nurse for 22 years. If I apply to the Minister of Health in Poland for a work permit, will I be allowed to work as a nurse or a massage therapist?
		- “Good evening. I have a diploma as a feldsher and I have worked in hospital for a total of about 22 years. I worked in dentistry for 8 years, in intensive care for 8 months, in traumatology for 3 years and cardiology for 10 years. I sent my documents to the ministry and they said that I have a medical degree, but I do not have at least 1 year of practice as a doctor, so they can’t hire me. Can I work in a private clinic as a nurse or dental assistant?
IV. Advertisements from employment agencies (*n* = 192)	11. Assistance in finding work in Poland	- Do you have a medical degree and are looking for a job in Poland? Fill in the form and we will contact you https://forms.gle/hxQkdab8spwv4oQw9 P.S.: Our services are free of charge, you sign the employment contract directly with the hospital/ clinic
		- Good morning, yes. Please call us for more information. We have a large selection of vacancies and we can assist you in obtaining a permit from the ministry and a licence
		- Good morning, we have an offer at the clinic in Miejska Górka in the Wielkopolskie Voivodeship. The fee is from 150 PLN/hour. We will help with all the documents to the Ministry. If you are interested, please feel free to contact us by priv
	12. Assistance in finding work abroad	- A healthcare recruitment company based in the UK is looking for people from Ukraine who would like to work in the UK. We are looking for doctors of all specialties, nurses, carers and caregivers for the elderly, and dentists. If you have a medical degree or want to study in this field, please contact us for more details. The basic requirement is language skills. You must be able to speak and write English to at least B2 level. All suitable candidates will receive a tailored package depending on their qualifications and the position sought. All suitable candidates will be provided with support/assistance throughout the immigration process. If you are qualified or want to work in the healthcare sector UK, please contact us to find out how we can help you
V. Seeking assistance related to problems in the daily life of war refugees from Ukraine in Poland (*n* = 47)	13. Seeking social support (e.g., seeking teaching aids for students)	- A fund-raising event for free laptops for Ukrainian students has been announced
	14. Seeking medical or psychological assistance (e.g., help in finding a doctor, assistance in accessing medication)	- Good morning! This post may be a bit off topic, but I need help. A lady friend, Lyudmila, who has left Dnipro has a dad with a heart condition - he has been left in Ukraine, where there are. More terrible shortages of medication. I would be very grateful for help with a prescription for two medications, Plavix 75 mg and Xarelto 20 mg. Many thanks in advance!
		- Good evening! Please let me know if I can benefit from a free appointment with a neurologist, I have been diagnosed with multiple sclerosis and at what address, if any
VI. Developing professional qualifications (*n* = 37)	15. Interest in language courses	- A friend from Ukraine is looking for a Polish for medical purposes language course. Can anyone give advice on where to look for such a course
	16. Interest in medical studies	- I want to study nursing in Poland. Where do I start? From first-cycle studies? What is the difference between first- and second-cycle studies? You cannot transfer to second-cycle studies without completing first-cycle studies, can you? What are the fees? I am not interested in a permit from the Minister of Health
		- Are there any nurses or feldspersons (medical assistants) from Ukraine who are studying nursing again? Are there any Ukrainian courses that have been credited and grades transcribed?

UKR-MP, Ukrainian Refugee background Medical Professionals; MH, Ministry of Health.

An external reviewer/expert with expertise in qualitative research, who was not involved in data collection or analysis, examined and validated the research protocol, data collection tools, extracted codes and themes.

### Ethical Considerations

The study did not require the approval of the Bioethics Committee of the Medical University of Warsaw due to the research methodology used and the nature of the data analysed [22, 23].

## Results

### Data Overview

Table 1 shows all categories included in the codebook, with six categories and sixteen subcategories of challenges for UKR-MPs entering the Polish healthcare system job market and examples of comments from our database (Table 1). Exemplary comments present selected, most frequent examples of challenges.

## Discussion

A review of the international literature has revealed a clearly insufficient number of publications on refugee background medical workers. The few available research papers on refugee background medical workers taking up employment only deal with the group of refugees arriving in Australia [24-27]. Furthermore, the available literature tends describe only those refugee background medical workers who have been residing in host countries for many years, often in refugee camps, obtaining both primary and secondary general education and vocational training in host countries [24-27].

The unique character of the analysis presented in our study makes it impossible to compare its results with those of other studies.

The UKR-MPs were expected to arrive in Poland after Russia’s invasion of Ukraine. Most of them received immediate assistance from Polish citizens and assistance from the state [28, 29]. The MH data show that 2,300 refugee doctors and 1,000 refugee nurses benefited from the simplified procedure for obtaining work permits, and the number of UKR-MPs interested in taking up employment in Poland is on the increase [30].

The most common problems encountered by UKR-MPs in this respect were: 1) lack of easy-to-understand information about the list of documents necessary to apply for a work permit, 2) lack of feedback from those responsible for handling individual cases, and 3) long waiting time for the decision issued by the MH.

As for the list of required documents, it should be noted that all the necessary information, updated on a regular basis, is available in Ukrainian on the MH website. Yet, for many years not Ukrainian but Russian had been the official language in Ukraine. It was only in 2017 that the use of Ukrainian became mandatory in most state institutions; nonetheless, a high percentage of Ukrainians have continued speaking Russian on a daily basis. This was particularly true in eastern Ukraine, and it was from this part of the country that the first wave of refugees residing in Poland arrived. This factor might have affected UKR-MPs ability to understand information published in Ukrainian on the MH website. Also, document templates are now available online that were not yet available in early 2022.

Content analysis also suggests that many applications were submitted by refugees not eligible to work as doctors or nurses in Poland but highly motivated to do so, e.g., applying for the position of a nurse with a feldsher’s degree obtained in Ukraine or applying for the position of an ultrasound specialist despite the fact that such a specialisation does not exist in Poland.

The refugees’ knowledge about the Polish educational system and the work setting was by far insufficient, which may have constituted an objective difficulty when trying to undertake work. In Ukraine, there still exists the profession of a feldsher, which may be acquired by graduating from a nursing secondary school or college of further education [31]. In Poland, with the abolition of feldsher education in the early 1960s, the profession tended to be omitted in legislation [32].

Poland implemented the Bologna Process standards, and with the accession to the EU in 2004, it also adopted the European Parliament and Council Directive 2005/36/EC, which regulates the recognition of professional qualifications for all EU member states. In accordance with it, the education system for the regulated professions, i.e., the professions of doctor and nurse, should be uniform and enable citizens to be mobile and take up work in any of the EU member states [33]. As Ukraine is not an EU member, these legal regulations do not apply within Ukraine, and training in the medical profession differs significantly from the European standards.

Bridging programmes for Polish nurses were just one example of the implementation of EU requirements. In the years 2008–2015, bridging courses were implemented from the European Social Fund for persons working in the nursing profession, but with only a secondary education, in order to supplement their qualifications in accordance with EU requirements. These programmes were successfully completed by over 37,000 nurses in Poland [34]. Concurrently, the educational system for nurses in Poland was being modified. Ukraine will be obliged to take similar measures. As an EU member state with experience in the necessary pre-accession activities, Poland could become Ukraine’s most important partner in this area.

The analysed Facebook posts showed a lack of UKR-MP knowledge of the existing discrepancies or a lack of awareness that such discrepancies exist. The refugees had neither been planning to change their work setting, nor had they been interested in the regulations in other countries. This unawareness may have caused UKR-MPs with a degree in pharmacy or that of a feldsher to apply for work as nurses, indicating that they had worked in this profession in Ukraine for many years. This hypothesis may also be indirectly confirmed by the data obtained from the MH Department for Medical Personnel Development. As for the group of nurses, by 12 September 2022, 618 applications had been submitted, of which 456 were approved (73%). In the following 3 months, until December 2022, the number of applications submitted amounted to 1,190, of which 761 were approved (63%). Fewer approvals probably resulted from an increasing number of applications submitted by refugees not eligible to work in the Polish healthcare system. The exact reasons for the refusals would require an in-depth analysis of the documentation.

Among UKR-MPs, the Polish language proficiency also poses a significant challenge, even though this aspect has been greatly liberalised. Prior to the 2022 armed conflict, the knowledge of the Polish language was a key criterion in the assessment of a foreigner entering the medical profession. The simplified work permit procedure no longer requires a language certificate, and the responsibility for employing a person with language proficiency appropriate for the job lies with the employer. This has raised a great deal of controversy among Polish nurses [35]. In view of the shortage of nurses in many healthcare institutions, and the urgent need to employ them, often in order to keep a given institution running smoothly, such a solution may cause concerns related to patient and staff safety, as well as the quality of medical services provided.

The analysis showed UKR-MP concerns regarding the level of proficiency in spoken and written Polish required by Polish employers. Refugees pointed to the fact that a Polish language certificate is required of doctors but this prerequisite does not apply to nurses. The findings of other researchers show that the knowledge of the language of the host country is the most important aspect of successful professional adaptation for refugees who wish to take up employment and it enables them to adapt more quickly to living in a country where they take up employment [26, 36].

Insufficient feedback from the MH on the status of one’s application was yet another issue raised by UKR-MPs, e.g., 1) difficulty contacting the MH Department for Medical Personnel Development, responsible for issuing work permits, either by e-mail or over the phone; 2) long waiting time for a final decision (around 6 months or longer); 3) lack of information on factors affecting the time taken to process individual applications; 4) an ineffective system of requests to supplement necessary documents. Furthermore, UKR-MPs did not receive information from the MH on the further steps required to obtain a work permit or the necessity to report on this matter to the District Chamber of Nurses and Midwives or the District Chamber of Physicians.

The occurrence of the aforementioned barriers may have stemmed both from factors attributable to the ineffective work of the public administration in Poland, as well as from a lack of experience in handling official matters in the host country by UKR-MPs. Despite the substantial changes implemented over the past year in terms of informing the UKR-MPs about the required documents, the implementation by the MH of an electronic document processing system should be undertaken to further facilitate this process. The MH is based in Warsaw and UKR-MPs are required to submit paper versions of documents. As a result, UKR-MPs would often submit documents by post, as they reside in remote parts of Poland. The need to process the documents without the support of an electronic system would certainly cause delays in processing an increasing number of applications. In addition, a shortage of administrative staff has been observed in the Polish healthcare system for several years [37]. The MH Department for Medical Personnel Development only employs three people and the number of administrative staff has not been increased to accommodate for the exponentially increasing number of applications [38]. On the other hand, there is also a risk that due to insufficient understanding of the requirements of the Ministry of Health (e.g., language barrier and lack of understanding of the system functioning), UKR-MPs often submitted inaccurate applications, burdening the administration of the Ministry of Health with the inappropriate documents and thus increasing the waiting time for a decision. The implementation of an electronic system for submitting applications is one example of how these barriers could be eliminated. Such a solution would also allow UKR-MPs to have an ongoing access to the status of the application, which in turn would reduce the need to contact the MH.

It now seems indispensable to make widely available comprehensive, practical information about undergraduate and postgraduate education and the requirements for taking up employment in the healthcare system in Poland. In our opinion, the group of healthcare beneficiaries is highly diverse, so such information could be made available by medical universities educating doctors and nurses, professional self-government bodies, or the Medical Centre for Postgraduate Education responsible for postgraduate education of medical professionals in Poland. These diverse entities, responsible for different stages of the preparation of medical personnel for work in Poland may cooperate to prepare comprehensive information on the functioning of the medical and nursing professions in Poland, available to UKR-MPs, given the fact that this group will be increasingly numerous.

Only a small number of the posts discussed courses developing professional or language competences, information on the possibility of studying in Poland, or assistance with daily activities of refugees in Poland. Insufficient knowledge of the Polish language preventing participation in training courses may be the most important factor behind this phenomenon. Furthermore, the vast majority of the refugees declare their willingness to return to Ukraine after the war [39]. Nonetheless, it should be emphasized that new professional skills obtained in Poland would improve the quality of medical services provided on return to Ukraine. Refugees arriving in Poland faced war trauma and challenging socio-economic conditions. Initially, their top priority was gaining work permits and securing employment rather than pursuing additional training to enhance their skills. Moreover, since this group had not yet begun work, their understanding of the competencies employers sought was limited. With an increase in experience and professional autonomy, their interest in enhancing their qualifications may grow, particularly among those intending to settle in Poland for good.

Also, it is important to add that war refugees, being one of the most vulnerable groups, are at risk of experiencing negative mental health outcomes such as posttraumatic stress disorder, anxiety, and depression [40-42]. This phenomenon is particularly severe in women [43] and children [44, 45]. In the group of Ukrainian war refugees staying in Poland, depression, anxiety disorders and PTSD may be observed in 73% of respondents, whereas 66% of the respondents display psychological distress [46, 47].

These conditions may be further exacerbated by a lack of social support due to language barriers and severed social connections with relatives, friends, and colleagues. These circumstances significantly hinder their ability to adapt to new situations and acquire new skills, knowledge and ability to cope with new challenges and obstacles while job seeking in the host country [48].

### Strengths

This first study to address the issue of obtaining work permits and taking up employment by UKR-MPs in Poland may serve as a valuable source of information on obstacles to access to the healthcare job market. It may form the basis for immediate practical corrective measures. The analysis of content created by UKR-MPs constitutes research material of particular significance. The analysis covers a year of observation, thus it reflects the changes and evolution of the described phenomena over time. The freedom of expression in social media in absence of the investigator, helps to obtain the users’ genuine opinions.

### Limitations

One significant limitation of the research results presented in this paper is the inability to provide an accurate socio-demographic description of the study group. This limitation arises from the fact that current technical restrictions on data retrieval from Facebook prevent obtaining socio-demographic information, following the data breach conflict between Facebook and Cambridge Analytica Data, c.f. a paper by Tommaso Venturini & Richard Rogers (2019) [49]. Though we are aware of these constraints, the analysis of Facebook content is nonetheless important to understand the above-mentioned challenges and to bridge a gap in current knowledge, particularly when it comes to issues affecting hard-to-reach groups of respondents, such as refugees.

### Further Research

The analysis of UKR-MPs needs as regards the functioning of the healthcare system in Poland and forms of the most effective and cost-efficient provision of further education, as expected by this group.

### Conclusion

Despite the promptly implemented administrative solutions enabling access to the job market by UKR-MPs, the refugees have encountered considerable administrative difficulties, as well as those arising from insufficient knowledge of the regulations on working as doctors and nurses in Poland under the EU law. The presented research also identifies the most urgent needs of UKR-MPs as regards supplementing their knowledge on the functioning of the healthcare system in Poland. Addressing these needs as quickly as possible may significantly affect the UKR-MP preparedness for work in Polish healthcare institutions, which will be beneficial for everyone.
